# Does curvilinear sprint training with different radii improve multidirectional speed and sprint mechanics in youth soccer players?

**DOI:** 10.5114/biolsport.2026.157996

**Published:** 2026-02-06

**Authors:** David Solleiro-Duran, Jose Suarez-Rial, Ezequiel Rey, Andrés Baena-Raya, Miguel Lorenzo-Martínez, Alberto Filter, Alexis Padrón-Cabo

**Affiliations:** 1 University of A Coruña, Department of Physical and Sports Education, Faculty of Sport Sciences and Physical Education, A Coruña, Spain; 2 Sport Sciences Department, RC Celta, Vigo, Spain; 3 University of Vigo, Faculty of Education and Sport Sciences, Pontevedra, Spain; 4 Instituto de Investigación en Ciencias del Deporte (CIDEGA), University of Vigo, Spain; 5 Department of Education, Faculty of Education Sciences, University of Almería, Almería, Spain; 6 SPORT Research Group (CTS-1024), CIBIS (Centro de Investigación para el Bienestar y la Inclusión Social) Research Center, University of Almería, Almería, Spain; 7 FSI Lab, Football Science Institute, Granada, Spain; 8 Research Group Physical Activity, Health and Sport CTS-948, Pablo de Olavide University, Sevilla, Spain

**Keywords:** Curve sprinting, Force-velocity profile, Change of direction, Sprint training, Multidirectional speed

## Abstract

This randomized parallel-group study examined the effects of curvilinear sprint (CS) training with different radii on linear sprint (LS) performance, CS ability, change of direction (COD), and horizontal force-velocity (FV) profile in youth soccer players. Eighteen male youth soccer players from the same academy were randomly assigned to a narrow CS group (NCSG; 5.15 m radius) or a wide CS group (WSCG; 11.15 m radius). Both groups completed 12 CS sessions over 6 weeks (2 sessions/week), integrated into their regular team training. Pre- and post-intervention assessments included LS (5–30 m), CS tests on both the good and weak sides over two radii (7.15 m and 9.15 m), modified 505 COD test, and sprint mechanical variables from the FV profile (F_0_, V_0_, P_max_, RF_max_, D_rf_). No significant time × group interactions were found for any variable (p > 0.05), indicating that narrow and wide CS training produced similar adaptations. However, within-group analyses revealed significant improvements (p ≤ 0.05) in most CS tests (except for the good side on 9.15 m), in 5 m and 20 m LS times and COD performance. Additionally, the NCSG and WCSG showed small to moderate significant enhancements (p ≤ 0.05) in P_max_ and RF_max_ after the training intervention. In conclusion, 6 weeks of CS training improved LS, CS ability, COD performance, and key sprint mechanical outputs in youth soccer players. These findings suggest that CS training is a specific and effective method for enhancing multidirectional speed and sprint mechanics in this population.

## INTRODUCTION

Soccer is an intermittent team sport characterized by frequent bouts of high-intensity accelerations, decelerations, and short sprint running, which are interspersed with longer periods of low-intensity activity [1]. Recent data from elite competitions, including the English Premier League and Spanish La Liga, show a notable increase in high-intensity running distances in recent years [2, 3]. Over the course of a match, players typically execute about ~91–119 highintensity accelerations (> 2.5–3 m·s^−2^) and ~16–27 sprints (> 6.6–7 m · s^−1^), highlighting the mechanical and neuromuscular demands of the game. Specifically, sprinting plays a crucial role in soccer, emerging as the most frequent individual action preceding goal situations [4]. Therefore, optimizing these high-intensity actions in soccer players should be considered a priority in the physical preparation of soccer players.

Importantly, sprinting in soccer is not limited to linear trajectories [5]. Recent research has revealed that around 70%-85% of the sprinting actions in male professional soccer players involve curvilinear trajectories [6, 4]. Typically within a radius of 3.5 to 11 m [7], curvilinear sprint (CS) is defined as “the upright running portion of the sprint completed with the presence of some degree of curvature” [8] and it presents unique biomechanical and neuromuscular characteristics compared to linear sprinting (LS) [9, 10]. Specifically, CS induces asymmetrical loading patterns between the inner and outer legs. Electromyographic analyses reveal increased activation of the adductors and semitendinosus in the inner leg, while the gluteus medius and biceps femoris are more active in the outer leg [10]. In terms of joint kinematics, the inner leg shows greater ankle eversion, hip adduction, and hip external rotation, along with longer ground contact times and lower resultant ground reaction forces when compared to the outer leg [11].

Sprinting training has emerged as a pivotal strategy in developing physical capabilities, sport-specific performance, and injury prevention in soccer [12, 13]. The sprinting ability of an athlete is effectively characterized through the horizontal force-velocity (FV) profile, which integrates individual mechanical outputs such as theoretical maximal force (F_0_), velocity (V_0_), and maximal power output (P_max_) during LS. This method also quantifies the maximal ratio of force applied in the forward direction at the sprint start (RF_max_), and the players’ ability to maintain net horizontal force production despite increasing sprint running velocity (D_rf_) [14]. Given the multidirectional nature of soccer, novel training strategies, such as curve sprinting using different radii, have emerged among strength and conditioning coaches aiming to better replicate match demands [15, 7, 16]. Recently, Ribić et al. [17] analyzed the mechanical demands of CS and reported that narrower radii reduce maximal theoretical speed while increasing maximal theoretical acceleration derived from the acceleration–speed profile in youth soccer players. These findings indicate that the mechanical and neuromuscular constraints of CS vary with curve radius. Specifically, narrow radii increase centripetal force requirements, producing higher mediolateral ground reaction forces and greater reorientation of the resultant force vector [18]. Likewise, it also increases neuromechanical stress and accentuates inter-limb force and loading asymmetries, whereas wider radii attenuate these constraints, resulting in force-application patterns closer to linear sprinting [19]. Thus, manipulating curve radius alters the mechanical demands of the task and may lead to different neuromuscular adaptations. However, it remains unknown whether sprint radius influences curvilinear sprint performance or horizontal force–velocity mechanical variables.

Therefore, the aim of this study was to analyze the effects of two curvilinear sprint training (CST) programs with different radii (narrow *vs*. wide) on the CS and linear performance, horizontal FV profile and change of direction (COD) ability in young soccer players. Based on previous evidence [17] we hypothesize that narrow CST would induce greater positive effect in CS performance and Wide CST would provide greater improvements in different LS distances and horizontal FV profile.

## MATERIALS AND METHODS

### Experimental approach to the problem

**FIG. 1:**
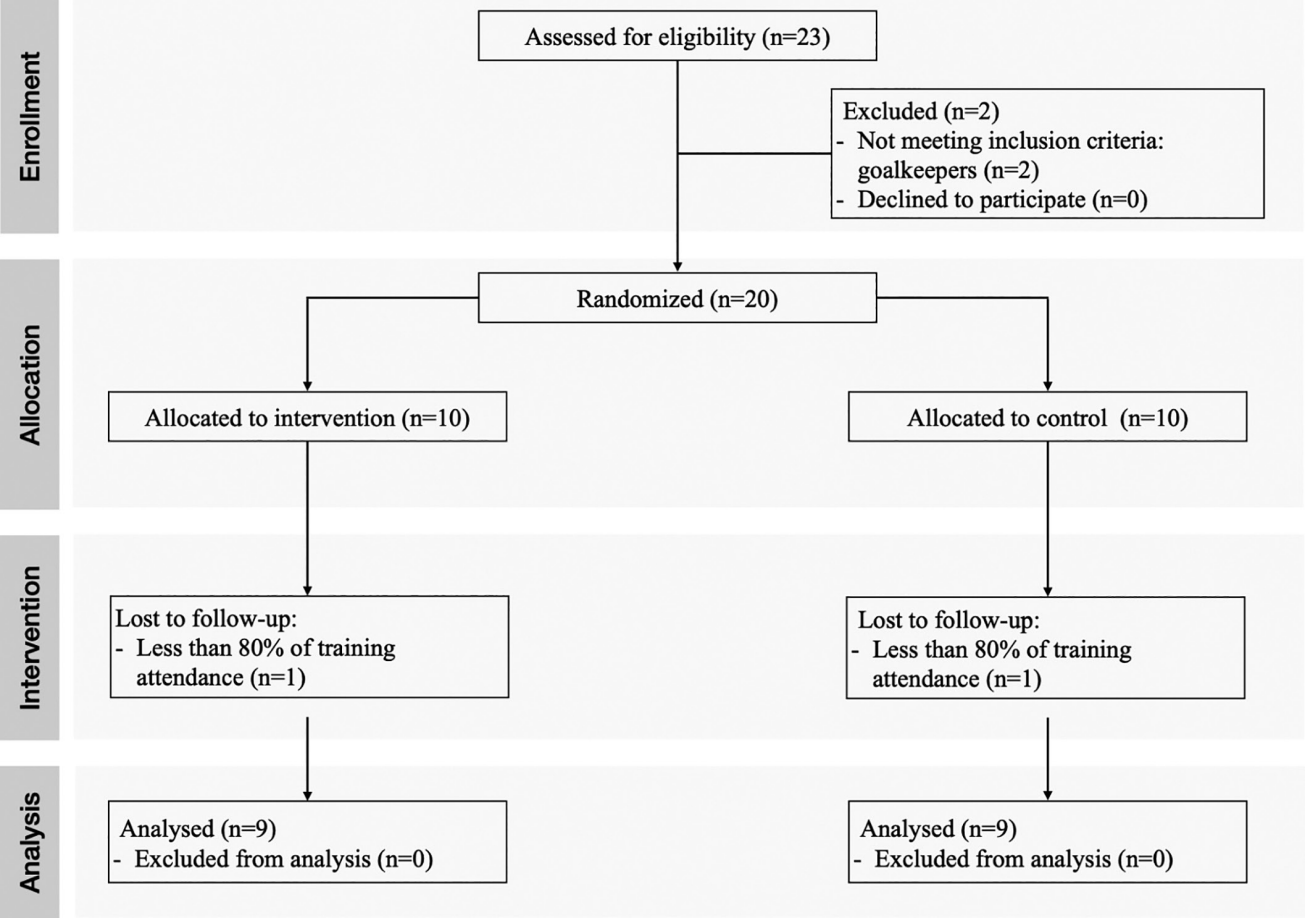
CONSORT diagram showing the flow of participants through each stage of a randomized trial.

A randomized pre-post parallel group trial design was performed to assess the effect of 6 weeks of a narrow or wide CS intervention on LS, CS, COD performance, and horizontal FV profile in youth soccer players. To avoid the risk of bias, a computer-generated sequence was employed for randomization. Following this procedure, participants were divided into the narrow curve sprint group (NCSG) or the wide curve sprint group (WCSG). Through the mid-season 23/24 period, the sprint intervention programs were incorporated into their standard soccer training regimen. One familiarization session was conducted prior to the baseline testing sessions to ensure participants were familiar with the testing protocol (Figure 1). The following multidirectional speed tests were performed: (a) 5 m sprint, (b) 10 m sprint, (c) 15 m sprint, (d) 20 m sprint, (e) 30 m sprint, (f) CS test 9.15 m, (g) CS test 7.15 m, and (h) modified 505 test. To minimize the impact of confounding variables, participants were instructed to maintain their lifestyle and typical dietary habits both before and throughout the intervention period. Furthermore, to mitigate the influence of fatigue, all tests were conducted after a 72-hour recovery period following an official match or intense training session. Both baseline and post-testing sessions were conducted during the same time window to minimize the impact of circadian rhythms and took place on an artificial grass surface and under similar environmental conditions. Participants were instructed to wear the same sports equipment across all testing sessions.

### Participants

**FIG. 2:**
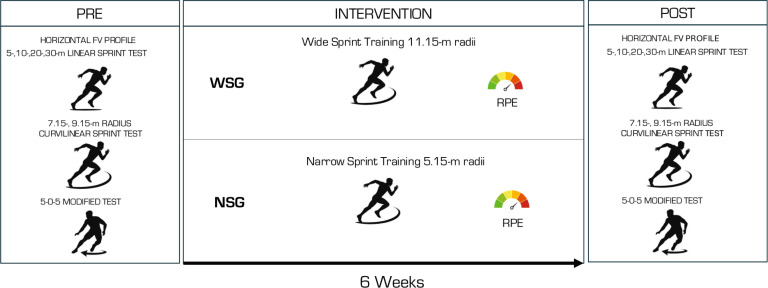
Schematic representation of the experimental protocol. Note: WCSG: wide curvilinear sprint training group; NCSG: narrow curvilinear sprint training group; FV: force-velocity.

Eighteen male youth soccer players from the same soccer academy club were recruited in this study. Prior to the recruitment procedure, a priori power analysis (G*Power, version 3.1.9.7, Universität Kiel, Düsseldorf, Germany) with an assumed Type I error of 0.05 and a Type II error rate of 0.20 (80% statistical power) was conducted for CS performance [20]. According to the prior power analysis for repeated measures ANOVA, a minimum sample size of eight subjects per group was estimated to be sufficient to detect medium group × time interaction effects (partial eta squared; η_p2_ = 0.125). As reported in the CONSORT flow (Figure 2), players were randomly assigned to NCSG (n = 9; age: 17.1 ± 0.7 years, height: 175.1 ± 5.7 cm; body mass: 68.8 ± 8.1 kg) or WCSG (n = 9; age: 16.8 ± 0.9 years, height: 173.3 ± 2.0 cm; body mass: 68.9 ± 8.8 kg). To participate in the current study, participants were required to meet the following inclusion criteria: (a) a minimum of four years of consistent training and competition experience in soccer and (b) absence of any injuries within the three months leading up to the intervention. Throughout the intervention phase, players engaged in three training sessions and one official match per week regularly. Moreover, the final statistical analyses were based solely on players who completed both familiarization and testing sessions, as well as attended at least 80% of all scheduled training sessions. All the subjects were informed of the risks and benefits of the study and both the players, and their parents or guardians gave their written consent before the initiation of the study. The research protocol and associated procedures were approved by the Local Ethics Committee (code: 04-280723). All procedures were conducted according to the Declaration of Helsinki for human studies [21].

### Testing Procedures

Before the pre-testing, one familiarization session was performed to minimize the potential learning effects. In this familiarization session, players were given instructions on the proper execution of each experimental test. Subsequently, the physical tests were conducted in two different sessions with 48 hours of rest between both, one week prior to starting the training intervention on the same artificial grass soccer pitch. Before the standardized warm-up at the start of the pre-test session, anthropometric measurements of the participants were conducted. Notably, the standing height was measured using a fixed stadiometer (Holstein Limited, Crosswell, United Kingdom), and body mass was measured to the nearest 0.1 kg using a digital scale (BC-554 Ironman Body Composition Monitor, Tanita^®^, Illinois, USA). Subsequently, a standardized warm-up was performed, comprising 5 minutes of low-intensity running, 5 minutes of dynamic stretching, and brief progressive accelerations. Furthermore, supervisors encouraged participants to exert maximal effort during physical tests.

### Curve Sprint Test

Based on the methodology described by Filter et al. [8] and Altmann et al. [22], CS tests with radii of 9.15 m and 7.15 m were implemented, both previously established as reliable for assessing sprint performance along curved trajectories. Both CS tests were calculated over a 17 m curved path, derived via trigonometric analysis of the arc geometry. The 9.15 m curve corresponds to the arc of the penalty area on a standard soccer field. The standardized parameters for the 9.15 m CS included: (a) a radius of 9.15 m from the penalty spot, (b) a straight-line distance of 14.6 m between the start and end points of the curve, and (c) a central angle of 105.84° from the penalty spot. Similarly, the 7.15 m CS was defined with the following parameters: (a) a 7.15 m radius from the penalty spot, (b) covering a straight-line distance of 13.3 m from the starting point to the curve’s endpoint, and (c) creating an angle of 136.3° from the penalty spot. In both CS tests, sprint time was recorded using dualbeam photoelectric timing gates (Witty, Microgate, Bolzano, Italy) positioned at the start and end points of the curve.

Participants initiated each sprint from a staggered standing position, with the lead foot placed 1 m beyond the first timing gate, as outlined by Filter et al. [8]. Two maximal attempts were performed for each direction (left and right curves), with a standardized recovery period of five minutes between trials [23]. The direction in which the athlete achieved their fastest time was designated as the “good” side, while the slower direction was classified as the “weak” side, based on the best trial performance for each curve orientation. The ICC_3,1_ for the test-retest trials were 0.94 (95% CI 86–0.97), 0.90 (95% CI 0.79–0.96), 0.99 (95% CI 0.98–1.00), 0.94 (95% CI 0.87–0.94) for the CS 9.15 Good Side, CS 9.15 Weak Side, CS 7.15 Good Side, and CS 7.15 Weak Side, respectively.

### Linear Sprint Test and Horizontal Force Velocity Profile profiling

LS performance was assessed using a system of five pairs of dualbeam photoelectric cell (Witty, Microgate, Bolzano, Italy), placed at distances of 0, 5, 10, 20, and 30 m. Participants initiated each sprint from a standing position, placing their preferred lead foot 0.5 m behind the initial timing gate. Prior to testing, athletes performed a standardized sprint-specific warm-up consisting of three progressive 30 m sprints at 50%, 70%, and 90% of their self-perceived maximum effort, in accordance with the protocol described by Marcote-Pequeño et al. [24]. Following the warm-up, each athlete completed two maximal 30 m sprint trials, interspersed with four minutes of passive recovery. Standardized verbal encouragement (“sprint as fast as possible”) was provided before each attempt. The fastest 30 m sprint time was selected for subsequent analysis. Velocity-time data were collected at a sampling frequency of 46.9 Hz using a Stalker Acceleration Testing System (ATS) II radar device (Model: Stalker ATS II Version 5.0.2.1; Applied Concepts, Dallas, TX, USA), which has been shown to be valid and reliable for assessing sprint-related variables [25]. The radar was affixed on a tripod positioned 10 m behind the starting line, aligned at a height of 1 m to approximate the athletes’ center of mass. Participants started each sprint from a crouched, staggered stance. The velocity-time data were subsequently used to derive individual sprint mechanical profiles, including F_0_, V_0_, and P_max_, following the field-based method proposed by Samozino et al. [14]. Both F_0_ and P_max_ were normalized to body mass to account for inter-individual variability. The ICC_3,1_ for the test-retest trials were 0.85 (95% CI 0.69–0.93), 0.85 (95% CI 0.67–0.93), 0.97 (95% CI 0.93–0.99), and 0.96 (95% CI 0.92–0.98) for the 5 m, 10 m, 20 m, and 30 m linear sprint respectively.

### Modified 505 Test

COD performance was assessed using a modified version of the 505 test (M505), with timing recorded via a dual-beam photoelectric cell system (Witty, Microgate, Bolzano, Italy), following the protocol described by Ryan et al. [26]. Participants commenced the test from a standing position, placing their preferred lead foot 0.5 m behind the initial timing gate. Upon receiving an auditory start signal, athletes sprinted forward through the timing gate, reached the 5 m turning line, executed a 180° COD using their dominant leg, and then sprinted back through the same gate over the final 5 m. To ensure protocol adherence, a supervisor was positioned at the turning point to verify correct execution. Trials in which the COD was initiated before reaching the designated line were considered invalid and repeated following a standardized recovery period. Each CS participant completed three valid trials, with three minutes of passive recovery between efforts. In addition to total test time, the change of direction deficit (COD_def_) was calculated as the difference between the M505 time and the 10 m LS time, in accordance with the method proposed by Nimphius et al. [27]. This metric provides a more isolated evaluation of COD ability by minimizing the influence of LS and acceleration capacities. The ICC_3,1_ for the tests-retest trials were 0.75 (95% CI 0.53–0.88) for the M505 test.

### Training Program

The intervention was performed during the mid-season 23/24. After pre-testing, the 6-week CST protocol (a total of 12 sessions) was developed twice a week (Table 1). At least the recovery period was 48 hours between sessions and after the official match. Both groups performed the same sprint distance per repetition, session, and week, with the same recovery time between repetitions and sets. Furthermore, participants were encouraged to provide maximal effort (i.e., all-out mode) during each CST [28, 29]. Based on prior research, it has been found that young male athletes can consistently regulate their performance during high-speed forward running with reliability [30]. The main difference between the groups was the radius of the CS: NCSG performed all CS with a radius of 5.15 m, while the WCSG executed the CS with a radius of 11.15 m. To standardize the trajectory in both groups, all trigonometric calculations were performed, and the sprint trajectory of both groups was marked on the ground. To apply the same training stimulus to both legs (i.e., good and weak sides), players performed an equal number of repetitions on both sides (i.e., clockwise and anticlockwise paths). All sessions were conducted on the same artificial pitch turf. Prior to each intervention training session, participants executed a standardized warm-up routine identical to that of the training sessions. The players were instructed to give maximal effort in each training session. A certified strength and conditioning specialist supervised all training sessions to ensure that all warm-up activities and sprints were completed with the correct technique and with maximum effort. Additionally, internal load and exercise intensity were quantified using Foster’s 0–10 rating of perceived exertion (RPE) scale [31]. Each participant reported his RPE individually 30 minutes after each training session to minimize the influence of acute emotional or physical sensations and to reduce potential bias in the ratings [32]. All players were already familiarized with the use of the RPE scale, as it was routinely implemented as part of their regular training monitoring within the academy.

**TABLE 1:** Descriptive characteristics of the curvilinear sprint training program performed by both experimental groups.

Weeks	Sessions per week	Sets	Repetitions	Distance per repetition (m)	Total distance per training (m)	Total distance per week (m)	Recovery between repetitions (min)	Recovery between sets (min)
1	2	1	6	17	102	204	2	5

2	2	2	4	17	136	272	2	5

3	2	2	5	17	170	340	2	5

4	2	4	3	17	204	408	2	5

5	2	4	3	17	204	408	2	5

6	2	2 2	4 3	17	136 102	238	2	5

### Statistical analyses

The statistical analyses were performed using the statistical package SPSS for Macintosh (version 25.0; Armonk, NY: IBM Corp) and statistical software R version 4.2.3 (R Core Team, 2023). Descriptive statistics are reported mean ± standard deviation (SD). The normality of distribution was assessed through visual inspection of Q-Q plots and Shapiro-Wilk test. Likewise, the homogeneity of variances was assessed using Levene’s test. A 2 (Time: Pre vs. Post) × 2 Group: WCSG vs. NCSG) repeated measures analysis of variance (ANOVA) was conducted to examine the effect of CST radius on multidirectional speed performance and sprint mechanical parameters derived from horizontal FV profile. Specifically, the effect size (ES) for this statistical analysis was reported using the partial eta squared (η_p2_) and categorized as small (≥ 0.01), medium (≥ 0.059), and large (≥ 0.138) effects. Additionally, the Cohen’s *d* was calculated and interpreted according to Hopkins et al. [33]: trivial (d *<* 0.2), small (0.2 ≤ *d* < 0.6), moderate (0.6 ≤ *d < 1*.2), large (1.2 ≤ *d* < 2.0), and very large (≥ 2.0). Regarding RPE, differences between sprinting protocols were analysed using a Linear Mixed Model. Group (WCSG vs. NCSG) and session were included as fixed factors, while player identity was treated as a random effect to account for repeated measures. Furthermore, the within-session reliability for all physical fitness tests was determined using the ICC_3,1_ [33]. The significance level for all analyses was set at *p* ≤ 0.05.

## RESULTS

**FIG. 3:**
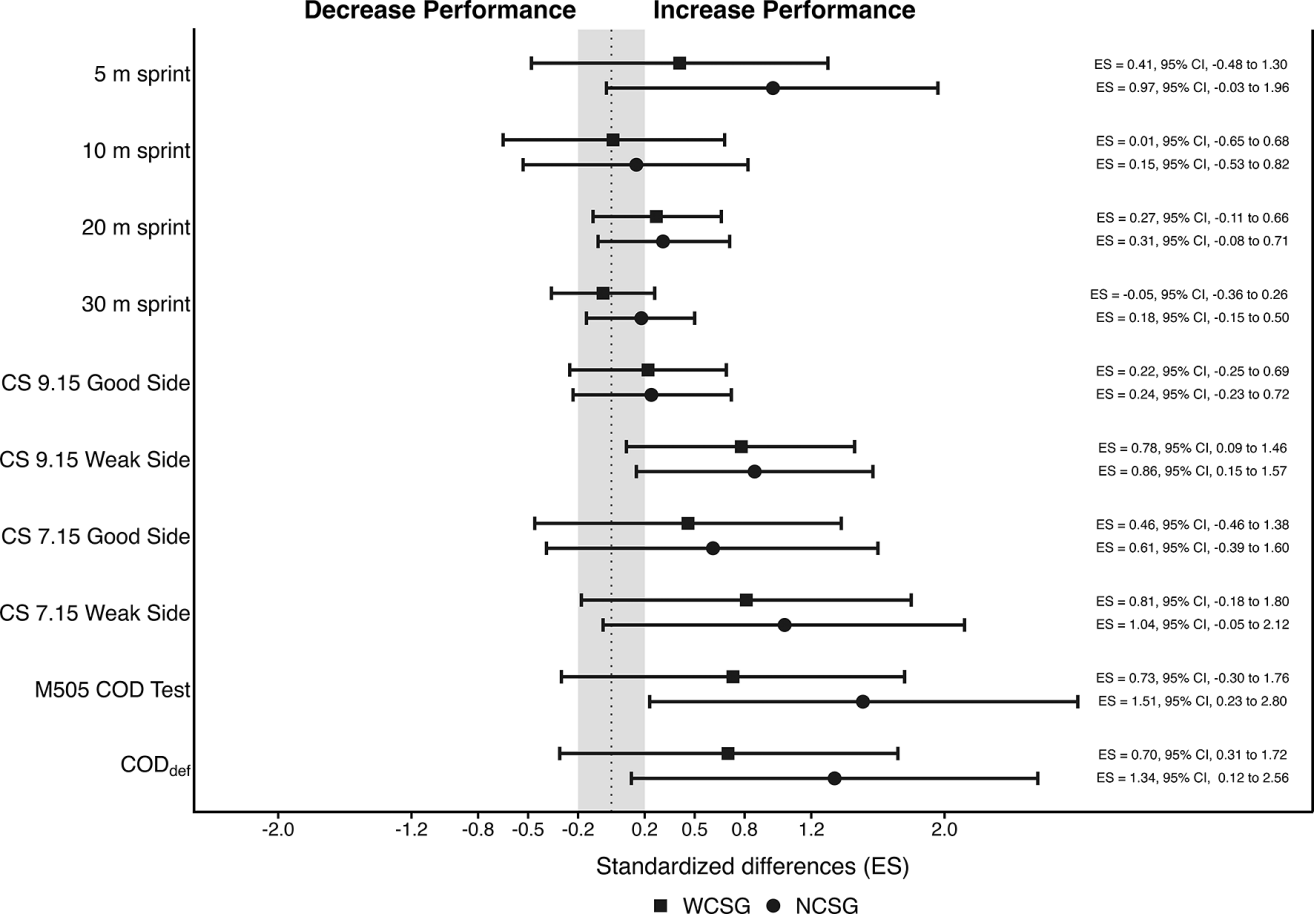
Standardized differences (95% CI) in all physical fitness variables between pre-and post-test for wide (filled squared) and narrow (filled circles) curvilinear training groups. Note: WCSG: wide curvilinear sprint training group; NCSG: narrow curvilinear sprint training group; ES: effect size; CS: curvilinear sprint; M505 COD Test; modified 5-0-5 change of direction test; CODdef: change of direction deficit.

The RPE values during the experimental training sessions were similar (*p* > 0.05) between NCSG (3.63 ± 1.53) and WCSG (3.14 ± 1.46). Mean values and SD, and percentage of change from pre- to post-intervention for linear speed, curvilinear speed, and COD tests are presented in Table 2. Additionally, Figure 3 displays the standardized mean differences between NCSG and WCSG from pre- to post-test.

**TABLE 2:** Changes in physical fitness after six weeks of CS sprint training in youth soccer players.

	WCSG (n = 9)			NCSG (n = 9)			ANOVA P values (*η*_p_^2^)		

	Pre	Post	∆ (%)	Pre	Post	∆ (%)	Time	Group	Time × Group
**5 m sprint** (s)	1.13 ± 0.05	1.11 ± 0.02	-1.42	1.13 ± 0.02	1.09 ± 0.05	-3.65	0.005 (0.396)	0.684 (0.011)	0.208 (0.097)
**10 m sprint** (s)	1.88 ± 0.09	1.88 ± 0.06	0.05	1.89 ± 0.07	1.88 ± 0.06	-0.52	0.630 (0.015)	0.813 (0.004)	0.687 (0.010)
**20 m sprint** (s)	3.25 ± 0.14	3.22 ± 0.11	-1.03	3.24 ± 0.13	3.20 ± 0.13	-1.23	0.003 (0.426)	0.762 (0.006)	0.821 (0.003)
**30 m sprint** (s)	4.47 ± 0.23	4.48 ± 0.20	0.27	4.49 ± 0.18	4.45 ± 0.20	-0.80	0.405 (0.044)	0.928 (0.001)	0.147 (0.127)
**CS 9.15 Good Side** (s)	2.78 ± 0.12	2.75 ± 0.13	-0.93	2.79 ± 0.11	2.76 ± 0.11	-1.02	0.051 (0.217)	0.861 (0.002)	0.910 (0.001)
**CS 9.15 Weak Side** (s)	2.84 ± 0.13	2.75 ± 0.13	-1.49	2.86 ± 0.11	2.76 ± 0.11	-2.30	< 0.001 (0.703)	0.811 (0.004)	0.761 (0.006)
**CS 7.15 Good Side** (s)	2.84 ± 0.15	2.78 ± 0.13	-2.07	2.84 ± 0.15	2.75 ± 0.10	-2.82	0.031 (0.273)	0.816 (0.004)	0.749 (0.007)
**CS 7.15 Weak Side** (s)	2.90 ± 0.15	2.79 ± 0.11	-3.58	2.94 ± 0.17	2.79 ± 0.10	-4.62	< 0.001 (0.532)	0.741 (0.008)	0.621 (0.017)
**M505 COD Test** (s)	2.67 ± 0.10	2.61 ± 0.09	-2.38	2.73 ± 0.07	2.59 ± 0.09	-4.90	< 0.001 (0.587)	0.607 (0.018)	0.128 (0.148)
**COD**_def_	0.79 ± 0.09	0.73 ± 0.09	-7.53	0.83 ± 0.08	0.70 ± 0.10	-14.51	< 0.001 (0.548)	0.909 (0.001)	0.202 (0.106)

Note: WCSG: wide curve sprint group; NCSG: narrow curve sprint group; CS 9.15 Good Side: curvilinear sprint 9.15 good side; CS 9.15 Weak Side: curvilinear sprint 9.15 weak side; CS 7.15 Good Side: curvilinear sprint 7.15 good side; CS 7.15 Weak Side: curvilinear sprint 7.15 weak side; M505 COD Test: modified 5-0-5 change of direction test; COD_def_: change of direction deficit.

**FIG. 4:**
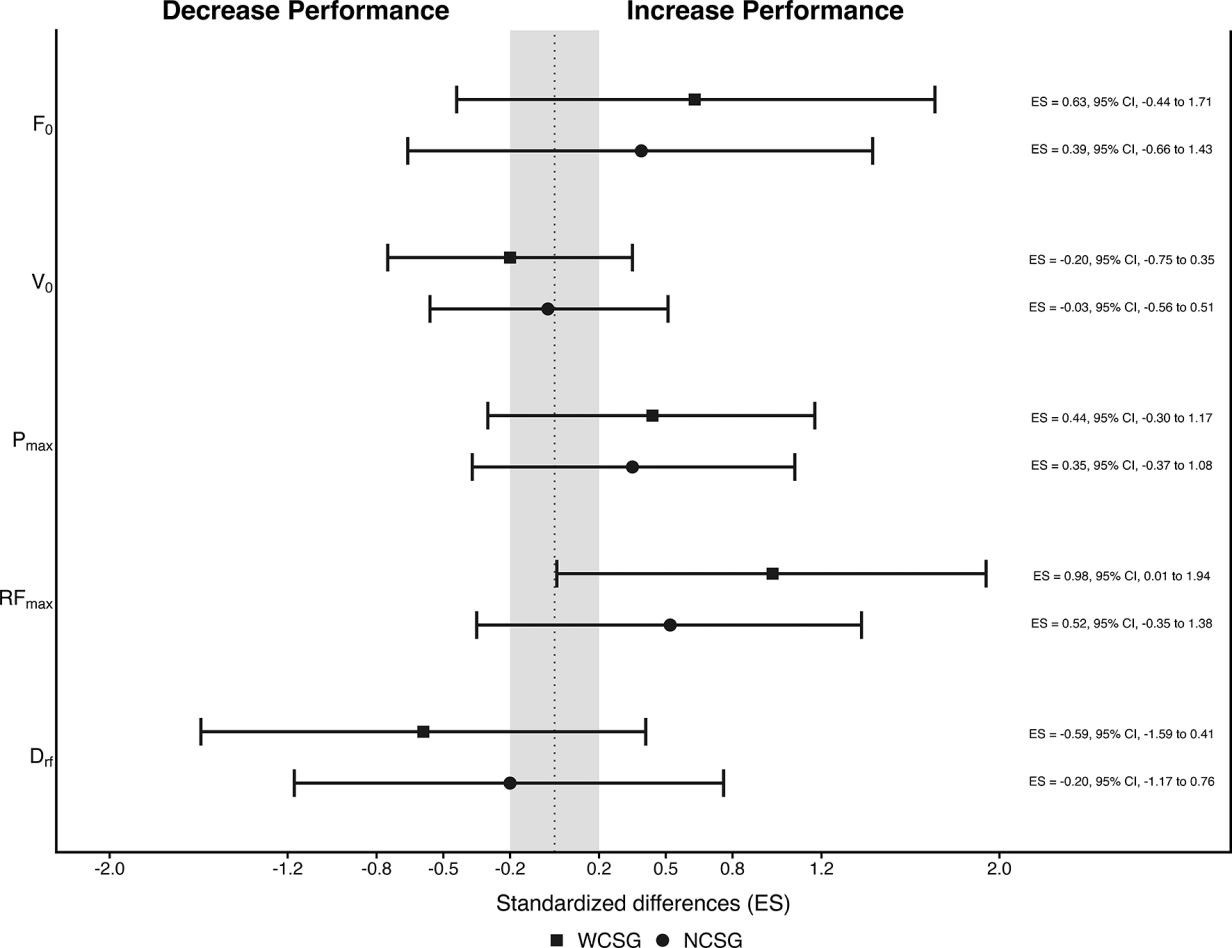
Standardized differences (95% CI) in horizontal FV profile variables between pre-and post-test for wide (filled squared) and narrow (filled circles) curvilinear sprint training groups. Note: WCSG: wide curvilinear sprint training group; NCSG: narrow curvilinear sprint training group; ES: effect size; F0: maximal theoretical horizontal force; V0; maximal theoretical running velocity; Pmax: maximal power output; RFpeak: proportion of total force production in the forward direction; DRF: decrease in the ratio of force with increasing running speed.

The results of horizontal FV profile parameters and standardized mean differences are presented in Table 3 and Figure 4, respectively.

**TABLE 3:** Changes in horizontal F-V profile after six weeks of sprint training in youth soccer players.

	WCSG (n = 9)			NCSG (n = 9)			ANOVA *P* values (*η*_p_^2^)		

	Pre	Post	∆ (%)	Pre	Post	∆ (%)	Time	Group	Time × Group
F_0_ (N/kg)	6.22 ± 0.57	6.65 ± 0.62	7.57	5.90 ± 0.59	6.17 ± 0.90	4.65	0.063 (0.200)	0.164 (0.117)	0.634 (0.015)
V_0_ (m/s)	8.54 ± 0.77	8.41 ± 0.73	-1.49	8.60 ± 0.57	8.59 ± 0.47	0.01	0.389 (0.047)	0.694 (0.010)	0.500 (0.029)
P_max_ (W/kg)	13.11 ± 1.23	13.84 ± 1.36	5.97	12.58 ± 1.58	13.16 ± 2.29	4.27	0.035 (0.250)	0.418 (0.041)	0.808 (0.004)
RF_max_ (%)	45.05 ± 2.89	48.11 ± 2.35	7.07	44.14 ± 3.66	45.75 ± 3.45	3.83	0.002 (0.458)	0.237 (0.086)	0.273 (0.074)
D_rf_ (%)	-6.70 ± 1.08	-7.23 ± 1.19	8.56	-6.30 ± 0.68	-6.53 ± 1.00	4.89	0.114 (0.149)	0.152 (0.124)	0.428 (0.040)

Note: WCSG: wide curvilinear sprint group; NCSG: narrow curve sprint group. F_0_: maximal theoretical horizontal force; V_0_: maximal theoretical running velocity; P_max_: maximal power output; RF_max_: proportion of total force production in the forward direction; D_rf_: decrease in the ratio of force with increasing running speed.

## DISCUSSION

This study analyzed the effects of two six-week CST programs, differing in curve radius (narrow vs. wide) but matched in training volume, on linear and CS performance, COD ability, and horizontal FV profile in young soccer players. The main findings of this study indicate that both training protocols led to significant improvements in 5 m and 20 m LS performance, as well as in key sprint mechanical variables such as P_max_ and RF_max_. Similarly, both groups showed significant enhancements in CS performance, with the exception of the good side in the 9.15 m CS test. Finally, performance in the M505 test and COD_def_ improved significantly following both training interventions. Collectively, the findings suggest that CS training is a specific and effective strategy for enhancing multidirectional speed and sprint mechanics in youth soccer players, although no statistically significant radius-specific effects were detected.

Time-motion analysis has shown that most sprinting actions in soccer follow curvilinear trajectories, covering a wide range of radii between 3.5 and 11 meters [16]. Recent evidence supports the effectiveness of CST in enhancing CS performance through trajectoryspecific adaptations, as compared to LS training [34]. However, to the best of the authors’ knowledge, this is the first study to examine how different curve radii influence CS performance. We found that both training protocols produced moderate improvements across CS tests, with the exception of the good side in the 9.15 m CS test. These findings align with those of Solleiro-Durán et al. [34], who reported moderate improvements in 9.15 m CS performance after six weeks of CST with a 9.15 m radius in U16 soccer players (good side ES = 1.05; weak side ES = 0.90), using similar sprint distances and training volume. Notably, the present study demonstrates that both narrow and wide radius CST can elicit meaningful improvements in CS performance, even when the training and testing radius do not fully coincide. In support of this, the strong correlation observed between the 7.15 m and 9.15 m CS tests suggests that improving CS ability may rely more on training CS itself than on matching the exact radius. However, as proposed by Chang et al. [19], tighter curves may place greater demands on joint stabilizer musculature to maintain efficient force production. Additionally, recent match-play analyses have shown that CS is the predominant sprinting pattern, often performed with tactical intentions such as closing down, overlapping, or recovering defensively [4, 6]. Although the present study did not include match-running or tactical-performance data, future research should examine whether improvements in CS ability translate to enhanced performance during match-deciding actions.

Sprint performance is widely recognized as a determinant action during soccer games [4]. This underscores the importance of developing sprint ability early in long-term athlete development [35]. While systematic reviews have suggested that primary training methods (i.e., sprint drills and unresisted sprints) produce limited improvements in sprint performance [36, 37], recent findings in youth soccer challenge this perspective. For instance, Rey et al. [28] reported significant short-distance sprint performance of up to −6.6% following targeted interventions. In addition, a recent study from Solleiro-Durán et al. [34], observed small to moderate significant improvements in 10 m, 20 m and 30 m LS performance after 11 CST sessions using a 9.15 m radius. By contrast, although the LS group significantly improved V_0_, P_max_ and RF_max_, the CS group improved only in P_max_ from pre-test to post-test, suggesting trajectory-specific adaptations in young soccer players. In the present study, both groups increased P_max_ and RF_max_, with no between-group differences in any of the FV profile parameters. These findings contrast with the principle of training specificity and the results of Morin et al. [38], who reported no significant improvements in the horizontal FV profile following LS training in adult male soccer players. Such discrepancies may be attributed to differences in performance level [39] and athletes’ training status, as non-elite players may exhibit greater responsiveness to training stimuli compared to elite players [40]. From a mechanical perspective, previous evidence demonstrated that sprinting on curves with larger radii facilitates higher running velocities and ground reaction forces [19], whereas narrower radii require greater centripetal acceleration to maintain the trajectory, resulting in higher mediolateral ground reaction forces and a more pronounced reorientation of the resultant force vector toward the center of the curve [18, 19]. These conditions increase neuromuscular asymmetry between legs, with the inside leg showing greater stabilizing and braking demands and the outside leg contributing more to propulsive force and hip external rotation [10]. However, these biomechanical and neuromuscular differences did not translate into differential sprint mechanical properties adaptations among youth soccer players.

CS has been conceptualized as an intermediate maneuver between LS and COD tasks with previous research reporting strong associations between CS kinematics and COD performance in soccer players [41]. In the present study, COD performance improved significantly in both training groups, with moderate to large effects observed in the WCSG (ES = 0.73) and NCSG (ES = 1.51), respectively. These results are consistent with previous findings from Solleiro-Durán et al. [34], who reported a 3.1% improvement in COD performance following six weeks of CST. Similarly, McMorrow et al. [42], observed enhancements of 3.3% and 3.7% in 180° COD tasks among professional soccer players after an in-season sprint training program that included both resisted and unresisted modalities. Improved COD ability is typically attributed to enhanced biomechanical efficiency and increased horizontal propulsive force [11, 43]. Although it could be hypothesized that narrower-radius CST may further improve COD performance due to its closer mechanical demands (greater contact times and force application during acceleration), no significant between-group differences were observed in this study. Furthermore, the present study demonstrated significant improvements in COD_def_ with reductions of 7.5% in the WCSG and 14.5% in the NCSG. These findings contrast with those reported by Solleiro-Durán et al. [34], who observed a nonsignificant change in COD_def_ despite a delta improvement of 4.1%. Collectively, these findings suggest that CST may enhance COD performance primarily through improvements in COD efficiency regardless of the CS radius [11], rather than through changes in LS mechanical variables such as F_0_ or V_0_.

These results highlight that integrating CST into regular training can simultaneously target speed, sprint mechanics, and agility without increasing total training time or sprint volume. Importantly, performance improved even when training and testing radii differed, suggesting that exposure to varied curvilinear trajectories may transfer across the range of curve demands encountered in competition. Coaches are therefore encouraged to incorporate CST with multiple radii within weekly microcycles to better replicate the biomechanical and neuromechanical demands of soccer. This approach may help athletes to maintain high velocities during curved runs, improve their efficiency in directional transitions, and ultimately enhance performance in match-deciding actions.

This study presents several limitations that must be acknowledged. First, the relatively small sample size (18 youth soccer players from a single academy) may limit the generalizability of the findings, as results might not translate to other academy contexts or competitive levels. In addition, although the sample size is comparable to previous applied training interventions in soccer, it may have been underpowered to detect subtle between-group differences, thereby increasing the risk of a Type II error due to inter-individual variability in sprint adaptations. Second, although a 6-week intervention is sufficient to induce performance improvements in youth players, it represents a relatively short period to optimize chronic training adaptations. Third, the absence of a non-training control group prevents isolating the specific effects of the CST intervention from other concurrent training stimuli. Moreover, maturation effects might also have influenced the training adaptations observed during the intervention period, although this factor was not considered in the analysis. Fourth, no kinetic or electromyographic data were collected, which limits our understanding of the neuromechanical mechanisms underlying the observed adaptations. Consequently, future studies should incorporate kinetic and electromyographic analyses to clarify how different curve radii influence force application and muscle activation strategies during curvilinear sprinting. Finally, future studies should assess ecological transfer to match play to determine whether improvements in sprint and COD performance translate into game-related outcomes.

## CONCLUSIONS

This research offers novel insights into the effects of CST with different radii in youth soccer players. Both narrow and wide CS interventions led to significant improvements in 5 m and 20 m LS times, performance weak-side on the 9.15 m CS, good- and weak-side performance on the 7.15 m CS, and key mechanical outputs from the horizontal FV profile (P_max_ and RF_max_). Likewise, the CS training demonstrated effectiveness in enhancing COD performance and reducing the COD_def_ in youth soccer players. Although both interventions resulted in meaningful performance improvements, no statistically significant were detected between radius conditions. In this regard, the comparable outcomes observed across groups suggest that the curve radius does not critically influence the magnitude of traininginduced adaptations. Rather, shared mechanical demands across CST modalities likely underlie the observed improvements in multidirectional speed qualities. Given the specific locomotor and mechanical demands of soccer, these findings support the inclusion of CST into youth soccer conditioning programs as a specific and efficient strategy to target sprint mechanics, asymmetry correction, and COD capabilities to effectively prepare players and enhance their linear and CS performance.
